# Visualization of a curated *Oryza sativa* L. CDPKs Protein-Protein Interaction Network *(*CDPK-OsPPIN*)*

**DOI:** 10.17912/micropub.biology.000513

**Published:** 2022-01-26

**Authors:** Joana Marques, Cleverson C. Matiolli, Isabel A. Abreu

**Affiliations:** 1 Instituto de Tecnologia Química e Biológica António Xavier, Universidade Nova de Lisboa (ITQB NOVA), Avenida da República, 2780-157 Oeiras, Portugal

## Abstract

Calcium-Dependent Protein Kinases (CDPKs) translate calcium ion (Ca^2+^) signals into direct phosphorylation of proteins involved in stress response and plant growth. To get a clear picture of CDPKs functions, we must identify and explore the CDPKs targets and their respective roles in plant physiology. Here, we present a manually curated *Oryza sativa* L. CDPK Protein-Protein Interaction Network (CDPK-OsPPIN). The CDPK-OsPPIN provides an interactive graphical tool to assist hypothesis generation by researchers investigating CDPK roles and functional diversity.

**Figure 1. Oryza sativa L. CDPKs Protein-Protein Interaction Network (OsPPIN):**
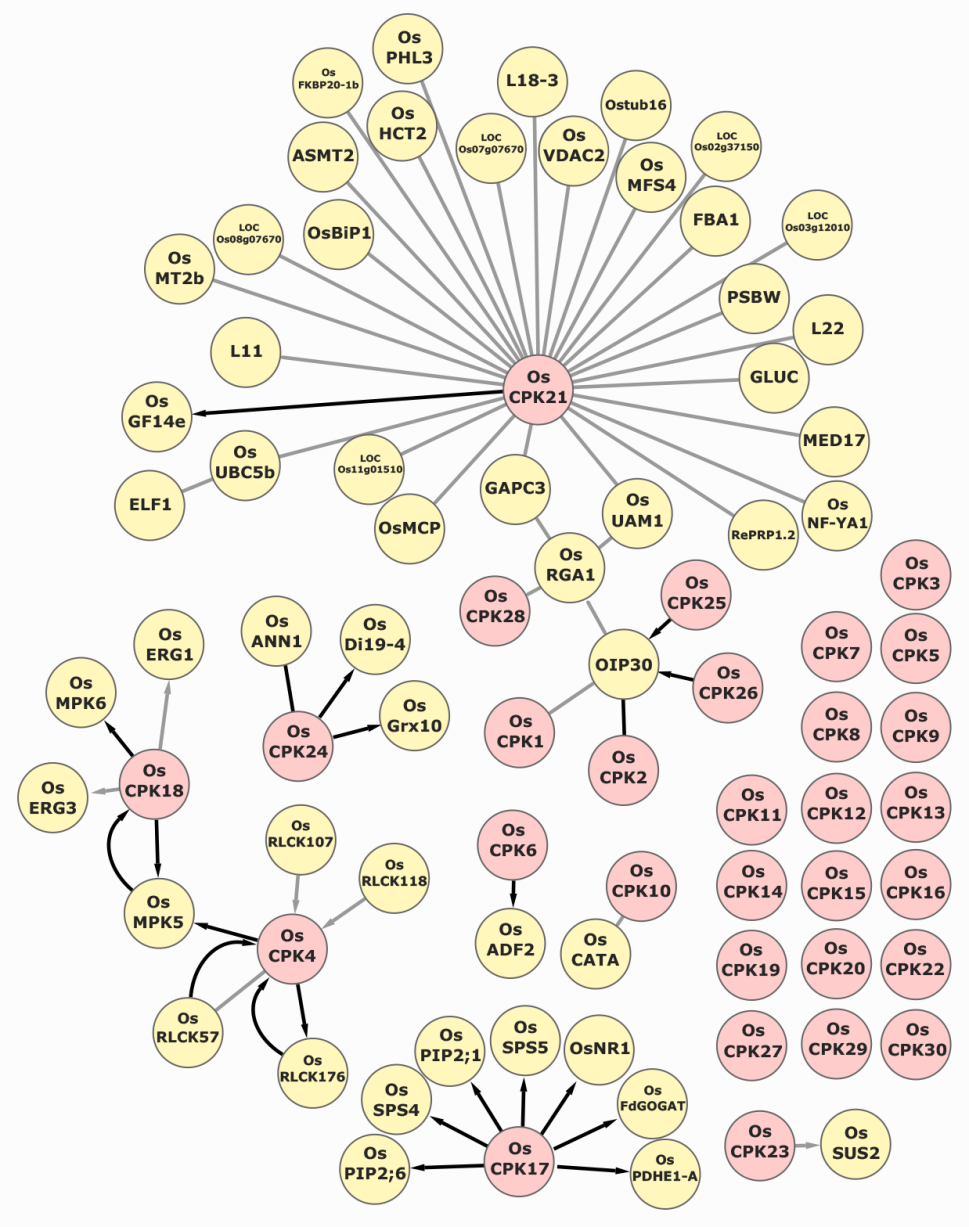
The network aggregates a manual curation of the rice CDPK-interacting proteins found in the literature and publicly available protein-protein interaction (PPI) databases (www.biogrid.org). The PPI assays supporting the interactions included in the OsPPIN comprise Yeast-Two-Hybrid (Y2H), Bimolecular Fluorescence Complementation (BiFC), Co-Immunoprecipitation (Co-IP), *in vitro* phosphorylation, *in vivo* phosphorylation, phosphoproteomics, interaction with target-specific peptides, and proteins identified by affinity capture followed by mass spectrometry. Edges connect CDPKs (in pink) and other proteins (in yellow); the arrow direction indicates phosphorylation targeting; edge color indicates the reliability of the PPI (gray or black, indicating information from one or more independent assays, respectively). The Gene/Protein names are the most commonly used in the literature. The detailed information about the CDPK OsPPIN network can be found: https://bit.ly/3205Tr9

## Description

Calcium-Dependent Protein Kinases (CDPKs) are essential translators of calcium ion (Ca^2+^) signaling in protists (Zhang and Choi, 2001), green algae (Valmonte *et al.*, 2014), and plants (Harmon *et al.*, 1987; Roberts, 1993). The ion Ca^2+^ is a key second messenger of diverse signaling pathways conveying environmental and developmental cues (Dodd *et al*., 2010). External and internal signals trigger transient changes of cytosolic Ca^2+ ^([Ca^2+^]_cyt_) levels. These changes in [Ca^2+^]_cyt_ levels can be perceived by Ca^2+^ binding proteins, such as Calmodulins (CaMs), Calmodulin-Like proteins (CMLs), Calcineurin B-Like proteins (CBLs), and CDPKs (Dodd *et al*., 2010). The perception of Ca^2+^ causes conformational changes in CaMs, CBLs, and CDPKs that allow specific protein-protein interactions. After Ca^2+^-induced conformational changes, CaMs interact with target proteins and recruit Calmodulin-dependent Protein Kinases (CaMKs) to phosphorylate the CaM targets (Zhang and Lu, 2003). Interestingly, CDPKs were proposed to result from a gene fusion between CaM and CaMKs genes (Zhang and Choi, 2001). Consequently, CDPKs are unique in their ability to sense and decode Ca^2+^ signals by directly phosphorylating specific targets.

CDPKs have a variable N-terminal domain, a serine/threonine kinase domain, and a regulatory calmodulin-like domain (CaM-LD). These kinases also possess an auto-inhibitory junction region that restrains their catalytic activity. The binding of Ca^2+^ to the EF-hands domains triggers a conformational change that exposes the kinase domain and activates the CDPK. The EF-hands of CDPKs vary in their affinity to Ca^2+ ^(Harmon *et al.*, 2000), suggesting that different CDPKs might respond to different Ca^2+^ concentrations. Ca^2+^ signals depend on the stimulus and differ in frequency of oscillation, amplitude, and duration (Dodd *et al.*, 2010). The timing of expression and spatial distribution of CDPKs can also add specificity to the Ca^2+^ signal decoding (Harmon *et al.*, 2000). However, how CDPKs determine the phosphorylation of their interacting proteins is still largely unknown. For instance, Wang *et al.*, 2011 showed that although both OsCPK2 and OsCPK26 interact with OsCPK25/26-Interacting Protein 30 (OIP30), only OsCPK26 can phosphorylate it.

CDPKs are involved in mediating plant stress responses. In rice (*Oryza sativa* L.), OsCPK4, OsCPK10, and OsCPK13 are involved in drought stress tolerance (Saijo *et al.*, 2000; Campo *et al.*, 2014; Bundó and Coca, 2017; Wang *et al.*, 2018). OsCPK4 and OsCPK10 enhance blast disease resistance (Bundó and Coca, 2016, 2017), while OsCPK12 and OsCPK18 seem to negatively regulate plant immunity (Asano *et al.*, 2012; Xie *et al.*, 2014). OsCPK12, OsCPK13, and OsCPK21 are involved in salt stress tolerance (Saijo *et al.*, 2000; Asano *et al.*, 2011, 2012), while OsCPK13 and OsCPK17 are involved in cold stress tolerance (Saijo *et al.*, 2000; Almadanim *et al.*, 2017). CDPKs also regulate central metabolism (recently reviewed in Alves *et al.*, 2021), suggesting that they can meditate growth-stress response balance in stressful conditions. However, to fully understand CDPKs’ function, mapping their interactions and phosphorylation targets is necessary.

Rice CDPKs are a large family of 30 members (Asano *et al.*, 2005; Alves *et al.*, 2021) still poorly characterized. Specifically, knowledge of their interactors, which define their function, is still scarce and lacking systematization. For instance, as of October 2021, BioGrid (v.3.5) reported 81,044 physical interactions in *Arabidopsis thaliana* but only 346 in rice – 236 of which result from a single experiment of affinity capture followed by mass spectrometry analysis (Stark *et al.*, 2006; Biswal *et al.*, 2019). Adding to this, the available information on rice-CDPKs can be challenging to retrieve due to the lack of standardization of gene nomenclature (see Material and Methods section). Working with rice CDPKs for over a decade, our lab felt the need to collect available functional information on CDPK interaction partners, systematizing it to make it readily available to the scientific community. A standard tool to organize and visualize Protein-Protein Interactions (PPI) is PPI Networks (PPIN). PPINs are a graphical representation and integration of large volumes of data and facilitates quick consults and the formulation of new hypotheses.

Here, we report the development of an *Oryza sativa* CDPKs PPIN (CDPK-OsPPIN, Figure 1). The interactive network can be found in (https://bit.ly/3205Tr9). The CDPK-OsPPIN will also be accessible on the web pages of the rice CDPKs of the RAP-DB (https://rapdb.dna.affrc.go.jp) (Kawahara *et al.*, 2013; Sakai *et al.*, 2013). The CDPK-OsPPIN provides a graphical and interactive interface to explore rice CDPK protein interactions and their involvement in rice signaling pathways coordinating plant fitness. To build the CDPK-OsPPIN, we compiled the literature available on the targets of OsCPKs. Additionally, we searched the interaction partners of the CDPK targets in the BioGrid repository (Stark *et al.*, 2006). The CDPK-OsPPIN was built using Cytoscape (v. 3.9.0) and is nested in the Ndex (v2.5.2) platform (https://www.ndexbio.org) (Shannon *et al.*, 2003; Pratt *et al.*, 2015). The Ndex platform allows for a searchable and interactive visualization of the CDPK repository. The nodes (proteins) are clickable and show the following information: Locus ID (MSU and RAP-DB) (Kawahara *et al.*, 2013; Sakai *et al.*, 2013), UniProt ID, other gene/protein symbols, molecular function, biological process, subcellular localization, and Gene Ontology (GO) terms. The interactions between proteins are represented by edges. The edges are also clickable and display the methodology used to determine each interaction and appear between the protein names (e.g., OsCPK21 (Y2H) PSBW) – empirically demonstrated phosphorylation (if tested), the phosphorylated residue (if applicable), and the literature reference that demonstrated the PPI with a clickable DOI. Phosphorylation is indicated by arrows on the edges. The PPIs were demonstrated by: Yeast-Two-Hybrid (Y2H) screenings, Bimolecular Fluorescence Complementation (BiFC), co-immunoprecipitation (Co-IP), *in vitro* phosphorylation, *in vivo* phosphorylation, protein identification by affinity capture followed by mass spectrometry, phosphoproteomic, and interaction with target-specific peptides. The number of experiments supporting each interaction is represented by the color of the edge, gray or black, indicating information from one or more independent assays, respectively. This representation allows the user to promptly infer the reliability of specific interactions – most PPIs represented in the network result from more than one low-throughput experiment. The protein and PPI metadata can also be retrieved in the ‘table format’ generated by the platform. The Ndex platform also allows queries that generate sub-networks. For instance, the query ‘nucleus’ will return a sub-network of nuclear proteins and their interactors. So far, CDPK-OsPPIN contains 89 proteins with 62 connections manually curated. The CDPK repository will be updated with manually curated data from future studies.

The CDPK-OsPPIN provides a graphical interface to facilitate hypothesis generation to explore the biological functions, functional divergence, and redundancy of CDPKs.

## Methods

The protein network is available at the Ndex platform (https://bit.ly/3205Tr9). The interaction data for the network were manually curated from the available literature. The protein-protein interaction network was built in Cytoscape (v. 3.9.0) (Shannon *et al.*, 2003). The nodes were displayed in organic layout. The protein annotations such as GO terms, subcellular localization, molecular function, and biological function were obtained from the Rice Genome Annotation Project (Kawahara *et al.*, 2013).

## Reagents

We adopted the following nomenclature when referring to a single member of the CDPK family is OsCPK*X*, where *X* refers to the number of the CPK. We suggest the adoption of this nomenclature by the scientific community. Here is the full list of members rice CDPK family, with the Rice Annotation Project Database (RAP-DB) Locus ID (Kawahara *et al.*, 2013; Sakai *et al.*, 2013). OsCPK1, Os01g0622600; OsCPK2, Os01g0808400; OsCPK3, Os01g0832300; OsCPK4, Os02g0126400; OsCPK5, Os02g0685900; OsCPK6, Os02g0832000; OsCPK7, Os03g0128700; OsCPK8, Os03g0808600; OsCPK9, Os03g0688300; OsCPK10, Os03g0788500 ; OsCPK11, Os03g0789000; OsCPK12, Os04g0560600; OsCPK13, Os04g0584600; OsCPK14, Os05g0491900; OsCPK15, Os05g0585500; OsCPK16, Os05g0467000; OsCPK17, Os07g0161600; OsCPK18, Os07g0409900; OsCPK19, Os07g0515100; OsCPK20, Os07g0568600; OsCPK21, Os08g0540400; OsCPK22, Os09g0514200; OsCPK23, Os10g0539600; OsCPK24, Os11g0171500; OsCPK25, Os11g0136600; OsCPK26, Os12g0133500; OsCPK27, Os12g0486600; OsCPK28, Os12g0169800; OsCPK29, Os12g0230200; OsCPK30, Os07g0641200.
